# Supplementary material for: Coral reproduction in Western Australia
Source: PeerJ. 2016 May 18;4:e2010. doi: 10.7717/peerj.2010 (PMC4878369; doi:10.7717/peerj.2010)
Supplement: Supplemental Information 1 [file peerj-04-2010-s001.pdf]

SUPPLEMENTARY TEXT 1. REGIONAL VARIATION IN ENVIRONMENTAL CONDITIONS ON WESTERN AUSTRALIAN REEFS

Kimberley Oceanic Region

The oceanic reefs in the Kimberley region (≈12˚S - 17.5 ˚S) of north-western Australia are isolated atolls rising from depths of several hundred meters near the edge of the continental shelf, more than 200 km from the coastline and from each other (Berry & Marsh 1986; Veron & Marsh 1988a; Wilson 2013). The dominant oceanic feature is the Indonesian Throughflow, which transports warm low salinity water into the region through the Indonesian Archipelago (Cresswell et al. 1993; Domingues et al. 2007; Holloway 1995). The Indonesian Throughflow also provides a mechanism for the transport of pelagic larvae from the Pacific and Southeast Asian reefs to north-western Australia, so the fauna at the oceanic atolls have a closer affinity to Indonesian reefs than to others in Western Australian waters. The local oceanography is influenced by semi-diurnal tides of up to approximately 5 m, and the waters around the reefs are warm, clear and nutrient deficient. Mean seawater temperatures vary seasonally, from a low of approximately 26° C in winter to maxima of approximately 30° C during December and March, with a small drop in mid-summer due to monsoonal cloud cover and storm activity.

The distance of the reef systems from the mainland means they are not affected by terrestrial runoff and associated issues of degraded water quality. There is also relatively little fishing pressure at the reefs, with the notable exception of traditional fishing by Indonesians for shark fin, *Trochus* and sea cucumber at some reefs (Stacey 2007), and/or some managed professional and recreational fishing. The most frequent disturbance to these reefs is seasonal storms and cyclones. Severe monsoonal winds and waves impact the reefs from a west, south-westerly direction between November and April, with cyclone impacts occurring through a similar period (Berry & Marsh 1986; Bowman et al. 2010). Since 1998, elevated water temperatures are known to have caused moderate to severe coral bleaching. Coral bleaching caused the mass mortality of corals at Scott Reef in 1998 (Gilmour et al. 2013), and less extreme temperature anomalies have caused moderate bleaching events at all the reefs during the last decade. Outbreaks of coral predators and disease have not been recorded, apart from a small outbreak of disease that affected some corals at parts of Scott Reef in 2010. However, most of the reproductive data collected from the oceanic atolls were not biased by these disturbances.

Kimberley Region

The Kimberley region (≈12˚S - 17.5 ˚S) of north-western Australia includes a vast network of fringing and coastal reefs, spanning over 4000 km of open coast and a coastline of over 13000 km (Short 2011; Wilson 2013). The oceanography is influenced by similar regional currents as the oceanic reefs, but with a more local influence of the Holloway Current adjacent to the shelf edge (D'Adamo et al. 2009; Wilson 2013). The local oceanography is dominated by a diurnal tidal range of up to 10 m, which coupled with strong currents and shallow bathymetry can cause periods of extreme turbidity on some reefs (Richards et al. 2014; Wilson 2013). Mean seawater temperatures are slightly higher than on the oceanic reefs, varying from approximately 25˚C in July to 31˚C in March. The coastal zone is characterised by extensive intertidal reefs, mudflats, mangrove forests, and muddy shorelines (INPEX 2011).

The remoteness of the Kimberley means it is currently not affected by degraded water quality or significant fishing pressure, although the region is of increasing economic importance and coastal developments are planned (Keesing et al. 2011). The most frequent disturbances to the region are monsoonal storms and cyclones through January to April (Veron & Marsh 1988a). There are no reports of outbreaks of coral disease or predators, or of temperature induced mass-bleaching events, but the remoteness of the region means there have been few surveys of the coral communities. However, the little reproductive data collected from the Kimberley region are unlikely to have been biased by disturbances.

Pilbara Region

The Pilbara region (≈19˚S - 21˚S) of Western Australia has extensive and varying coral reef habitats, ranging from highly turbid, sheltered reefs along the mainland to wave exposed fringing reefs on mid-shelf islands (Blakeway & Radford 2004; Veron & Marsh 1988b; Wilson 2013). The regional oceanography reflects a zone of transition from the dominant currents in the Kimberley region, particularly the Holloway Current, to the region in which the Leeuwin Current intensifies to the north of Ningaloo reef. As with the Kimberley, the longshore, wind-driven currents of the Pilbara region are linked to the seasonal changes in winter tradewinds and summer monsoonal storms, but are dominated locally by relatively

large (5 m) semi-diurnal tides and the complex topography of the mainland and island archipelagos (Wilson 2013). Water temperature within the inshore area has the largest seasonal range in Western Australia, of between 20 to 30° C, and can reach as high as 34° C in late summer (Jones 2004). High concentrations of plankton, organic detritus and particulate sediments result in high turbidity at the inshore reefs, which is further elevated by the shallow bathymetry, tidal range, strong currents, and resuspension of sediments by seasonal winds, storms and cyclones (Jones 2004).

The tropical, semi-arid climate within the Pilbara is punctuated by high temperatures, sporadic rainfall, and frequent tropical cyclones between December and April (Jones 2004). Damage from tropical cyclones is significant, causing some of the highest historical wind gusts recorded in Australia. Associated with these storms are the localised impacts of freshwater run-off from the mainland and islands. More recently, temperature induced coral bleaching has caused severe, and in places repeated, damage to Pilbara reefs. Coral bleaching and mortality was first observed in the Dampier Archipelago in 1998, and since 2008 coral bleaching has been reported on reefs adjacent to the mainland at Dampier and Onslow, in the Dampier Archipelago, and around Barrow and Montebello Islands (Chevron 2014; Moore et al. 2012; MScience 2010; Speed et al. 2013). Additionally, outbreaks of the coral eating snail (*Drupella cornus*) and particularly the crown-of-thorns starfish (*Acanthaster planci*) have been reported periodically on Pilbara reefs, and have caused severe impacts at some sites within the Dampier Archipelago (Wilson 2013). In addition to these more regional scale disturbances, some reefs within the Pilbara experience additional pressure from recreational fishing and industrial developments, particularly within Mermaid Sound and in the west Pilbara. Mermaid Sound is the site of a major industrial port servicing resource development in the northwest of Australia (Hanley 2011) and in the west Pilbara major gas developments with associated port infrastructure are located around the mainland at Onslow and Barrow Island. Considerable research and monitoring efforts have been carried out to document marine flora and fauna around these developments, with an emphasis on effects of elevated turbidity and sedimentation from dredging operations (e.g. Rosser & Baird 2009; Stoddart et al. 2012; Stoddart & Gilmour 2005). Many of the inshore coral reefs of the Dampier Archipelago are naturally turbid, with increases in turbidity and sedimentation in recent decades generated by port dredging, so the current coral assemblages are likely to be relatively tolerant to these conditions. In contrast, reefs in the outer archipelago are naturally less turbid, which is reflected in their coral assemblages. Port developments and dredging

operations have more recently altered water quality on reefs in the west Pilbara. The cumulative impacts of these operations and background impacts from cyclones and coral bleaching have in recent years reduced cover and diversity, increased susceptibility of assemblages to diseases, and reduced rates of coral growth, survival and recruitment (Chevron 2014; Pollock et al. 2014). These disturbances have biased at least some of the coral reproductive data collected from the Pilbara region.

Ningaloo Region

Ningaloo Reef (≈ 21.5˚S – 23.5˚S) is the largest continuous reef system in Western Australia. The reef system consists of approximately 300 km of barrier and fringing reefs (Veron & Marsh 1988a), with reef passages connecting the offshore waters to the extensive back-reef and lagoon. The dominant oceanographic feature in the region is the Leeuwin Current, which intensifies around Ningaloo Reef as the southward flow of warm water in autumn and winter (Cassata & Collins 2008; Holloway & Nye 1985), and disperses tropical larvae to sub-tropical reefs. However, this is countered by the northern flow of the Ningaloo Current adjacent to the coastline from spring to winter, a process that can produce upwelling events that cool the waters on the western side of Northwest Cape during the summer (Taylor & Pearce 1999; Woo et al. 2006). Relatively small, semi-diurnal tides of up to approximately 2 m have little influence on the regional oceanography. Mean monthly sea surface temperatures vary seasonally, from a low of approximately 23° C in late winter and early spring, to a high of approximately 28° C in late summer and the early autumn months. Ningaloo Reef has a unique fauna, but with some affinities to the Pilbara reefs to the north and those in the region of Shark Bay and the Abrolhos Islands to the south (Veron & Marsh 1988a). Affinities with Pilbara reefs are particularly strong for reefs such as Bundegi, located on the eastern side of Northwest Cape, which experience greater environmental variability and higher turbidity and sedimentation. The outer reef edge is surrounded by clear oceanic water and subjected to heavy wave action, with a strong flow of water over the reef flat.

There are no major industrial or port developments in the region of Ningaloo, and the lack of major river systems means the reefs are not affected by terrestrial runoff and the associated issues of degraded water quality. There are commercial fishing operations in the region and also recreational fishing pressure. One of the most common disturbances is severe storms and tropical cyclones, usually approaching from a north-westerly direction from November to

April. In recent years, recurrent temperature anomalies have also affected Ningaloo Reef, and in the summer of 2010-2011 parts of the reef suffered severe impacts from a combination of a coral bleaching and cyclone (Depczynski et al. 2013; Moore et al. 2012). There are few records of outbreaks of coral disease or the predatory crown-of-thorns starfish, but outbreaks of the coral-eating snail *Drupella* have been reported periodically (Black & Johnston 1994; Holborn et al. 1994). However, these disturbances are unlikely to have biased the existing data on coral reproduction for the region.

Abrolhos and Shark Bay Region

The Abrolhos Islands (≈28˚S - 29˚S) are the most southerly accretive reef formations in the Indian Ocean, comprising four island groups that are near the continental shelf edge (Veron & Marsh 1988a). Situated to the north of the Abrolhos Islands, Shark Bay (≈26˚S) is a large shallow bay (~12,950 km^2) enclosed by a number of islands, with an average depth of 9 m (Veron & Marsh 1988a). The bay consists of vast seagrass meadows (Wells et al. 1985) and localised coral communities, but few data on coral cover and community composition exist (but see Bancroft 2009; Cary 1997; Moore et al. 2011). The distribution and diversity of reefs in the region are probably facilitated by the Leeuwin current, which brings warm waters and coral larvae from the north during autumn and winter following the main period of spawning at Ningaloo Reef (Hatcher 1991; Hutchins & Pearce 1994; Taylor & Pearce 1999). Water temperatures during winter are therefore relatively warm, ranging seasonally between 20° C and 25° C (Pearce 1986; Veron & Marsh 1988b). The seaward reefs of the Abrolhos are exposed to strong wave action and have extensive areas of algal growth, whereas most coral communities occur in back reef areas and form patches in western lagoons (Veron & Marsh 1988a).

The reefs at Shark Bay and the Abrolhos Islands are not currently impacted by major industrial or port developments, nor terrestrial runoff, and typically have high water quality. Despite being located at relatively high latitudes, however, the flow of warm water with the Leeuwin Current has caused temperature anomalies and coral bleaching at the Abrolhos in 2011 (Abdo et al. 2012). The Abrolhos Islands have a targeted fishery of western rock lobster (*Panulirus cygnus*), the most valued single-species fishery in Australia (Caputi et al. 1996). The effect of the fishery and the removal of biomass on the ecosystem is inconclusive (Bellchambers 2010), but other commercial developments may have some impact on the

coral reefs in the region (e.g. Oceanica & MScience 2006). The limited existing data on coral reproduction at the Abrolhos Islands is unlikely to have been biased by disturbances.

Southwest Region

The South West region (≈30˚S - 33˚S) includes temperate reef patches around the Perth metropolitan area (e.g. Hall Bank and Marmion lagoon), at Rottnest Island 20km off the coastline, and 250km north of Perth in Jurian Bay. Hard corals are ubiquitous on the temperate limestone reefs along the coast of the region (Thomson et al. 2012), but with a mean cover of less than few percent (Speed et al. 2013). Areas where corals form more significant components of the benthic assemblages are rare and are restricted spatially, most commonly on the leeward side of islands or in bays and lagoons where they are sheltered from the westerly winds and waves generated by winter storms (Hatcher 1989; Veron & Marsh 1988a). The dominant oceanographic influence for the coral reefs around Rottnest Island is the southerly flow of warm tropical water through autumn and winter with the Leeuwin Current (Veron & Marsh 1988a). However, the Leeuwin Current has less influence on the reef patches and lagoons adjacent (< 3 km) to the Perth Metropolitan area, which experience lower water temperatures and higher turbidity due to terrestrial runoff (Harriott & Banks 2002; Thomson & Frisch 2010).

Marine habitats surrounding Rottnest Island and Hall Bank are exposed to extensive wave energy during winter storms (Wells & Walker 1993). Although rarely documented, coral bleaching due to high temperature anomalies has affected both shallow and deep water coral communities around Rottnest Island (Thomson et al. 2011). In addition, the corals in the metropolitan area are also exposed to pressures related to urbanization and infrastructure development, such as terrestrial runoff, dredging and port development, and recreational and commercial fishing (SKM 2011). However, the reproductive data available for the southwest region are unlikely to have been biased by disturbances.

Abdo DA, Bellchambers LM, Evans SN, and Evans S. 2012. Turning up the heat: Increasing temperature and coral bleaching at the high latitude coral reefs of the Houtman Abrolhos Islands. *Plos one* 7:e43878.

Bancroft KP. 2009. Mapping the coral reef communities of Shark Bay marine protected areas: data collected during the February 2008 field survey. Marine Science Program Data Report Series 3. Perth: Department of Environment and Conservation, Kensington, WA. p 45.

Bellchambers L. 2010. *The effect of western rock lobster fishing on the deepwater ecosystems of the west coast of Western Australia*: Fisheries Department of WA.

Berry PF, and Marsh LM. 1986. Faunal Surveys of The Rowley Shoals, Scott Reef and Seringapatam, NW Australia. Records of the Western Australian Museum, Supplement No 26. Perth.

Black R, and Johnston MS. 1994. Growth rates in outbreak populations of the corallivorous gastropod *Drupella cornus* (Röding, 1798) at Ningaloo Reef, Western Australia. *Coral Reefs* 13:145-150.

Blakeway D, and Radford B. 2004. Scleractinian corals of the Dampier Port and inner Mermaid Sound: species list, community composition and distributional data. *Corals of the Dampier Harbour: their survival and reproduction during the dredging programs of 2004*.

Bowman D, Brown G, Braby M, Brown J, Cook L, Crisp M, Ford F, Haberle S, Hughes J, and Isagi Y. 2010. Biogeography of the Australian monsoon tropics. *Journal of Biogeography* 37:201-216.

Caputi N, Fletcher W, Pearce A, and Chubb C. 1996. Effect of the Leeuwin Current on the recruitment of fish and invertebrates along the Western Australian coast. *Marine and Freshwater Research* 47:147-155.

Cary J. 1997. Baseline studies and monitoring of visitor sites in the Shark Bay Marine Park, Shark Bay World Heritage Area and Hamelin Pool Marine Nature Reserve, Shark Bay marine reserves monitoring programme: final report MMSP/MW/SBMP-4/1997. Perth: Commonwealth Department of Tourism and Department of Conservation and Land Management.

Cassata L, and Collins LB. 2008. Coral reef communities, habitats and substrates in and near Sanctuary Zones of Ningaloo Marine Park. *Coastal Research* 24:139.

Chevron. 2014. Gorgon Gas Development and Jansz Feed Gas Pipeline: Post-Development Coastal and Marine State and Environmental Impact Survey Report, Year 3: 2013-2014. Perth, Western Australia Chevron Australia Pty Ltd

Cresswell G, Frische A, Peterson J, and Quadfasel D. 1993. Circulation in the Timor Sea. *Journal of Geophysical Research: Oceans* 98:14379-14389.

D'Adamo N, Fandry C, Buchan S, and Domingues C. 2009. Northern Sources of the Leeuwin Current and the "Holloway Current" on the North West Shelf. *Journal of the Royal Society of Western Australia* 92:53-66.

Depczynski M, Gilmour J, Ridgway T, Barnes H, Heyward A, Holmes T, Moore J, Radford B, Thomson D, and Tinkler P. 2013. Bleaching, coral mortality and subsequent survivorship on a West Australian fringing reef. *Coral Reefs* 32:233-238.

Domingues CM, Maltrud ME, Wijffels SE, Church JA, and Tomszak M. 2007. Simulated Lagrangian pathways between the Leeuwin Current System and the upper-ocean circulation of the southeast Indin Ocean. *Deep-Sea Research Part II Topical Studies in Oceanography* 54:797-817.

Gilmour JP, Smith LD, Heyward AJ, Baird AH, and Pratchett MS. 2013. Recovery of an isolated coral reef system following severe disturbance. *Science* 340:69-71.

Hanley J. 2011. Environmental monitoring programs on recent capital dredging projects in the Pilbara (2003-2010): a review. *APPEA Journal* 2011:273-293.

Harriott VJ, and Banks SA. 2002. Latitudinal variation in coral communities in eastern Australia: a qualitative biophysical model of factors regulating coral reefs. *Coral Reefs* 21:83-94.

Hatcher A. 1989. Variation in the components of benthic community structure in a coastal lagoon as a function of spatial scale. *Marine and Freshwater Research* 40:79-96.

Hatcher BG. 1991. Coral reefs in the Leeuwin Current -- an ecological perspective. *Journal of the Royal Society of Western Australia* 74:115-127.

Holborn K, Johnston MS, and Black R. 1994. Population genetics of the corallivorous gastropod *Drupella cornus* at Ningaloo reef, western Australia. *Coral Reefs* 13:33-39.

Holloway PE. 1995. Leeuwin current observations on the Australian North West Shelf, May–June 1993. *Deep Sea Research Part 1: Oceanographic Research Papers* 42:285-305.

Holloway PE, and Nye HC. 1985. Leeuwin Current and wind distributions of the southern part of the Australian North West Shelf between January 1982 and July 1983. *Marine and Freshwater Research* 36:123-137.

Hutchins JB, and Pearce AF. 1994. Influence of the Leeuwin Current on Recruitment of Tropical Reef Fishes at Rottnest Island, Western-Australia. *Bulletin of Marine Science* 54:245-255.

INPEX. 2011. Biological and ecological studies of the Bonaparte Archipelago and Browse Basin. Perth: INPEX - Ichthys Project.

Jones DS. 2004. The Burrup Peninsula and Dampier Archipelago, Western Australia: an introduction to the history of its discovery and study, marine habitats and their flora and fauna. *Aquatic Fauna of the Waters of the Dampier Archipelago, Western Australia Report of the Woodside Energy Ltd/Western Australia Museum Partnership to Explore the Marine Biodiversity of the Dampier Archipelago* 66:27-49.

Keesing JK, Irvine TR, Alderslade P, Clapin G, Fromont J, Hosie AM, Huisman JM, Phillips JC, Naughton KM, and Marsh LM. 2011. Marine benthic flora and fauna of Gourdon Bay and the Dampier Peninsula in the Kimberley region of north-western Australia. *Journal of the Royal Society of Western Australia* 94:285-301.

Moore J, Bellchambers L, Depczynski ME, RD, Field S, Friedman K, Gilmour J, Holmes T, Middlebrook R, Radford B, Ridgeway T, Shedrawi G, Taylor H, Thompson D, and Wilson S. 2012. Unprecedented Mass Bleaching and Loss of Coral across 12° of Latitude in Western Australia in 2010–11. *Plos one* 7(12): e51807. doi:10.1371/journal.pone.0051807

Moore JAY, Bancroft KP, and Holley D. 2011. Coral reef communities. In Western Australian Marine Monitoring Program: annual marine protected area condition pressure response report: Shark Bay Marine Park and Hamelin Pool Marine Nature Reserve annual report, 2011. In: Bancroft K, editor. Perth: Department of Environment and Conservation, Kensington, WA. p 24-35.

MScience. 2010. Dampier Port upgrade dredging program 2006: Long term coral habitat monitoring & management August 2010. Perth.

Oceanica, and MScience. 2006. Abrolhos Resort Development Long Island Marine Monitoring and Management Plan. Perth.

Pearce A. 1986. Sea temperatures off Western Australia. *Fishing Industry News Service* 19:6-9.

Pollock FJ, Lamb JB, Field SN, Heron SF, Schaffelke B, Shedrawi G, Bourne DG, and Willis BL. 2014. Sediment and Turbidity Associated with Offshore Dredging Increase Coral Disease Prevalence on Nearby Reefs. *Plos one* 9:e102498.

Richards ZT, Sampey A, and Marsh L. 2014. Marine Biodiversity of the Kimberley 1880s–2009. Kimberley marine biota Historical data: scleractinian corals: Records of the Western Australian Museum Supplement 84. p 111-132.

Rosser NL, and Baird AH. 2009. Multi-specific coral spawning in spring and autumn in far north-western Australia. *Proceedings of the 11th International Coral Reef Symposium*.

Short AD. 2011. Kimberley beach and barrier systems: An overview. *Journal of the Royal Society of Western Australia* 94:121-132.

SKM. 2011. Fremantle Ports Inner Harbour Deepening and Reclamation – PostDredging Coral Monitoring Report. Perth: Sinclair Knight Merz.

Speed CW, Babcock RC, Bancroft KP, Beckley LE, Bellchambers LM, Depczynski M, Field SN, Friedman KJ, Gilmour JP, and Hobbs J-PA. 2013. Dynamic Stability of Coral Reefs on the West Australian Coast. *Plos one* 8:e69863.

Stacey N. 2007. *Boats to burn - Bajo fishing activity in the Australian Fishing Zone*. Canberra: Australian National University E Press.

Stoddart C, Stoddart J, and Blakeway D. 2012. Summer spawning of *Porites lutea* from north-western Australia. *Coral Reefs* 31:787-792.

Stoddart JA, and Gilmour J. 2005. Patterns of reproduction of in-shore corals of the Dampier Harbour, Western Australia, and comparisons with other reefs. In: Stoddart SE, ed. *Corals of the Dampier Harbour: their survival and reproduction during the dredging programs of 2004*. Perth: MScience Pty Ltd.

Taylor JG, and Pearce AF. 1999. Ningaloo Reef currents: implications for coral spawn dispersal, zooplankton and whale shark abundance. *Journal of the Royal Society of Western Australia*:57-65.

Thomson D, Babcock R, Vanderklift M, Symonds G, and Gunson J. 2012. Evidence for persistent patch structure on temperate reefs and multiple hypotheses for their creation and maintenance. *Estuarine, Coastal and Shelf Science* 96:105-113.

Thomson D, Bearham D, Graham F, and Eagle J. 2011. High latitude, deeper water coral bleaching at Rottnest Island, Western Australia. *Coral Reefs* 30:1107-1107.

Thomson D, and Frisch A. 2010. Extraordinarily high coral cover on a nearshore, high-latitude reef in south-west Australia. *Coral Reefs* 29:923-927. 10.1007/s00338-010-0650-1

Veron JEN, and Marsh LM. 1988a. Hermatypic corals of Western Australia. Records of the Western Australian Museum. ed. Perth, Western Australia. p 1-136.

Veron JEN, and Marsh LM. 1988b. Hermatypic corals of Western Australia: records and annotated species list. *Records of the Australian Museum* Supplement No. 29:1-133.

Wells FE, Rose R, and Lang S. 1985. An analysis of benthic marine invertebrate communities in subtidal seagrass and sand habitats in Shark Bay, Western Australia. *Records of the Western Australian Museum* 12:47-56.

Wells FE, and Walker DI. 1993. Introduction to the marine environment of Rottnest Island, Western Australia. *The Marine Flora and Fauna of Rottnest Island, Western Australia*: Western Australian Museum.

Wilson B. 2013. *The Biogeography of the Australian North West Shelf: Environmental Change and Life's Response*: Newnes.

Woo M, Pattiaratchi C, and Schroeder W. 2006. Summer surface circulation along the Gascoyne continental shelf, Western Australia. *Continental Shelf Research* 26:132-152.
