# Supplementary material for: Coral reproduction in Western Australia
Source: PeerJ. 2016 May 18;4:e2010. doi: 10.7717/peerj.2010 (PMC4878369; doi:10.7717/peerj.2010)
Supplement: Supplemental Information 2 [file peerj-04-2010-s002.pdf]

SUPPLEMENTARY TABLE 1. SOURCES OF CORAL REPRODUCTIVE DATA FOR REGIONS ACROSS WESTERN AUSTRALIA

Data were sourced from public journal articles and reports, plus confidential reports written by private consultants and provided by government and industry. Raw data were not available for all reports and text and derived data within reports were used to contribute to the regional summaries.

Region	Publication
**Kimberley Oceanic**	Gilmour, J., Smith L. D., et al. (2009). Biannual spawning, rapid larval development and evidence of self-seeding for scleractinian corals at an isolated system of reefs. *Marine Biology* 156: 1297-1309.<br>Gilmour, J. P., Travers M. J., et al. (2011). Long-term monitoring of coral and fish communities at Scott Reef. SRRP Project 1 Final Report for Woodside Energy as operator of the Browse LNG Development. Perth, Western Australia, Australian Institute of Marine Science. 157pp.<br>Gilmour J.P., Underwood J.N., Howells E.J., Gates E., and Heyward A.J. (2016). Biannual Spawning and Temporal Reproductive Isolation in *Acropora* Corals. *Plos one* 11:11(13): e0150916. doi:0150910.0151371/journal.pone.0150916.<br>Heyward, A. J., Jones, R. J. *et al.* (2012). Monitoring study S6B Coral Reefs, Montara: 2011 Shallow reef surveys at Ashmore, Cartier and Seringapatam Reefs. Final Report for PTTEP Australiasia (Ashmore Cartier) Pty. Ltd. Australian Institute of Marine Science, Townsville. 163pp.<br>Heyward, A. J., Radford. B. *et al.* (2013). Seringapatam Reef Baseline Surveys 2012-2013. Final Report for Conoco Philips (Browse Basin) Pty Ltd. Australian Institute of Marine Science, Perth 2013. 117pp.
**Kimberly Oceanic, Ningaloo**	Rosser, N. (2013). Biannual coral spawning decreases at higher latitudes on Western Australian reefs. *Coral Reefs* 32(2): 455-460.
**Kimberley**	INPEX (2011). Biological and ecological studies of the Bonaparte Archipelago and Browse Basin. Perth, INPEX - Ichthys Project.
**Kimberley, Pilbara**	Rosser, N. and Baird A. H. (2009). Multi-specific coral spawning in spring and autumn in far north-western Australia. *Proc 11th Int Coral Reef Sym.* 1:366-370.

Region	Publication
**Pilbara**	Baird, A. H., Blakeway D., *et al.* (2011). Seasonality of coral reproduction in the Dampier Archipelago, northern Western Australia. *Marine Biology* 158: 275-285.<br>Mscience (2007). Pluto LNG Development - Spawning Assessment October 2007, Report No. MSA93R3.<br>Mscience (2008). Pluto LNG Development - Spawning Assessment March 2008, Report No. MSA93R22.<br>Mscience (2009). Pluto LNG Development - Spawning Assessment February 2009, Report No. MSA93R78.
	Mscience (2009). Pluto LNG Development - Spawning Assessment March 2009, Report No. MSA93R85.<br>Mscience (2009). Pluto LNG Development - Spawning Assessment April 2009, Report No. MSA93R92.<br>Mscience (2009). Pluto LNG Development - Re-assessment of Spring 2008 Spawning in *Porites*, Report No. MSA93R104.<br>Mscience (2009). Pluto LNG Development - Porites Spawning Assessment – Summer 2009, Report No. MSA93R126.<br>SKM (2011). Cape Lambert Port B Development Dredging Marine Environmental Monitoring - Coral Spawning Assessment Summary Report - February Spawning Window, Rev 1 WV05051-FI-RS-001, March 2011.<br>SKM (2011). Cape Lambert Port B Development Dredging Marine Environmental Monitoring - Coral Spawning Assessment Summary Report - March Spawning Window, Rev 0 WV05051-FI-RS-0002, March 2011.<br>SKM (2011). Cape Lambert Port B Development Dredging Marine Environmental Monitoring - Coral Spawning Assessment Summary Report - October Spawning Window, Rev 0 WV05051-FI-RS-0003, October 2011.<br>SKM (2011). Cape Lambert Port B Development Dredging Marine Environmental Monitoring - Coral Spawning Assessment Summary Report - November Spawning Window, Rev 0 WV05051-FI-RS-0004, November 2011.<br>SKM (2011). Cape Lambert Port B Development Dredging Marine Environmental Monitoring - Coral Spawning Assessment Summary Report - December Spawning Window, Rev A WV05051-FI-RS-0005, December 2011.<br>SKM (2012). Cape Lambert Port B Development Dredging Marine Environmental Monitoring - Coral Spawning Assessment Summary Report - February Spawning Window, Rev 0 WV04505-FI-RS-0001, February 2012.<br>SKM (2012).Cape Lambert Port B Development Dredging Marine Environmental Monitoring - Coral Spawning Assessment Summary Report, March Spawning Window, Rev 0 WV04505-FI-RS-0002, March 2012.

Region	Publication
**Pilbara**	SKM (2010). Proposed Outer Harbour Development Port Hedland - Coral spawning spring surveys 2009 Revision B - WV03716-MV-RP-0041 04, June 2010.<br>SKM (2010). Proposed Outer Harbour Development Port Hedland - Coral spawning Autumn surveys 2009 Revision 0WV03716-MV-RP-0034 2, September 2009.<br>Simpson, C. J. (1985). Mass spawning of scleractinian corals in the Dampier Archipelago and the implications for management of coral reefs in Western Australia. Perth, Western Australia, Department of Conservation and Environment. 224: 35pp.<br>Stoddart, J. A. and J. Gilmour (2005). Patterns of reproduction of in-shore corals of the Dampier Harbour, Western Australia, and comparisons with other reefs. Corals of the Dampier Harbour: Their Survival and Reproduction During the Dredging Programs of 2004. S. E. Stoddart. Perth, MScience Pty Ltd. 53-64.<br>Stoddart C, Stoddart J, Blakeway D (2012). Summer spawning of *Porites lutea* from north-western Australia. *Coral Reefs* 31:787-792.<br>Rosser, N. L. and J. P. Gilmour (2008). New insights into patterns of coral spawning on Western Australian reefs. *Coral Reefs* 27(2): 1-5.
**Pilbara, Ningaloo**	Rosser, N. L. (2005). Reproductive seasonality and biannual spawning of *Acropora* on two north-west Australian reefs. Murdoch University. Western Australia. 125pp.
**Ningaloo**	Gilmour J. 1999. Experimental investigation into the effects of suspended sediment on fertilisation, larval survival and settlement in a scleractinian coral. *Marine Biology* 135:451-462.<br>Heyward A.J., Smith L.D., Rees M., and Field S.N. (2002). Enhancement of coral recruitment by in situ mass culture of coral larvae. *Marine Ecology Progress Series* 230:113-118.<br>Smith, R. A. (1993). Mass Spawning in the *Faviidae* on the Ningaloo Reef Tract. Perth, Western Australia, Murdoch University. 126pp.<br>Stewart, R. R. (1993). Mass Spawning in the *Acroporidae* on the Ningaloo Reef Tract. Science. Perth, Western Australia, Murdoch University. 94pp.<br>Taylor J.G., and Pearce A.F. (1999). Ningaloo Reef currents: implications for coral spawn dispersal, zooplankton and whale shark abundance. *Journal of the Royal Society of Western Australia*. 82: 57-65.

Region	Publication
**Abrolhos Islands**	Babcock, R. C., B. Willis, et al. (1994). Mass spawning of corals on a high latitude coral reef. *Coral Reefs* 13: 161-169.<br>Dinsdale E.A., and Smith L. D. (2004). Broadscale survey of coral condition on the reefs of the Easter Group of the Houtman Abrolhos Islands. Report the Western Australian Department of Fisheries. 23pp.<br>Foster T., Gilmour J.P., *et. al.* (2015). Effect of ocean warming and acidification on the early life stages of subtropical *Acropora spicifera*. *Coral Reefs*: 1-10.
**Southwest**	Crane, K. R. (1999). Reproductive Biology of Scleractinian Coral at Rottnest Island, Western Australia. Marine Department. Perth, Murdoch University: 160.
