# Supplementary material for: Coral reproduction in Western Australia
Source: PeerJ. 2016 May 18;4:e2010. doi: 10.7717/peerj.2010 (PMC4878369; doi:10.7717/peerj.2010)
Supplement: Supplemental Information 3 [file peerj-04-2010-s003.pdf]

SUPPLEMENTARY TABLE 2. REGIONAL VARIATION IN SPAWNING FOR CORAL SPECIES ON WESTERN AUSTRALIAN REEFS

Seasons and months in which coral species are thought to spawn based on the available data; evidence for some species is likely biased by issues with identification and methodology. Species do not spawn during all months below in every year, but have been inferred to spawn in the months below during one or more years. For example, the primary month of spawning on many reefs during autumn is March and during spring is October, but spawning can also occur in April and November due to split-spawning or a late full moon during some years. Similarly, within species an equal proportion of colonies do not spawn during each month. For example, in species that spawn during autumn and spring across Western Australia, a higher proportion of colonies typically spawn during autumn. Regions are colour coded according to Figure 1. Seasons and months are: Spring (Spr), September (s), October (o), November (n); Summer (Sum), December (d), January (j), February (f); Autumn (Aut), March (m), April (a), May (m). Spawning has not been recorded during Winter months (June, July, August) in Western Australia and they have been excluded. Species names are presented as per the original literature and taxonomic revisions are in Supplementary Table 3.

Based on the available data, the sampling design and the methods used, confidence in the inferred months of spawning was ranked qualitatively according to:

1 Confident. Evidence based on the presence of pigmented eggs in colonies prior to the predicted dates of spawning in many colonies, sites and years; the presence and absence of pigmented eggs in many colonies around the predicted dates of spawning; and/or direct observations of spawning in multiple colonies.

2 Likely. Evidence based on the presence of pigmented eggs in many colonies prior to the predicted dates but with limited spatial and temporal replication; and/or most evidence indicates spawning during this month but with some contradictory data among studies.

3 Possible. Evidence based on the presence of large but unpigmented eggs several weeks prior to the predicted dates of spawning; and/or contradictory data among studies due to sampling design, methodology or species identification.

4 Unlikely. No evidence of spawning; pigmented or large unpigmented eggs absent from samples of many colonies, sites and years within several weeks of the predicted dates of spawning.

Species	Kimberley Oceanic									Kimberley								
	Spr			Sum			Aut			Spr			Sum			Aut		
	s	o	n	d	j	f	m	a	m	s	o	n	d	j	f	m	a	m
Acanthastrea echinata																		
Acropora abrolhosensis		4	4				2	2										
Acropora aculeus		2	2				2	2										
Acropora acuminata							2	2										
Acropora anthocercis		2	2				1	1				2						
Acropora aspera							1	1				4				4		
Acropora austera		2	2				4	4				4						
nomen dubium																		
Acropora carduus							2	2										
Acropora cerealis		2	2				1	1				4						
Acropora clathrata		2	2				2	2				4						
Acropora cytherea		1	1				1	1				2						
Acropora abrotanoides																		
Acropora dendrum												4						
Acropora desalwii							2	2										
Acropora digitifera		3	3				1	1			2	2				2		
Acropora divaricata		4	4				2	2										
Acropora donei		2	2				2	2										
Acropora echinata																		
Acropora florida		1	1				1	1				4				2		
Acropora muricata																		
Acropora gemmifera	2	1	1				1	1				4						

Species	Pilbara									Ningaloo								
	Spr			Sum			Aut			Spr			Sum			Aut		
	s	o	n	d	j	f	m	a	m	s	o	n	d	j	f	m	a	m
Acanthastrea echinata		4					2											
Acropora abrolhosensis																		
Acropora aculeus																		
Acropora acuminata		4					3	3										
Acropora anthocercis							2									2	2	
Acropora aspera		4	4				1											
Acropora austera		4					3	3								4	4	
nomen dubium		4					2											
Acropora carduus		4					2											
Acropora cerealis							3	3			4	4					1	
Acropora clathrata							2											
Acropora cytherea		2														3	3	
Acropora abrotanoides							1											
Acropora dendrum																		
Acropora desalwii																		
Acropora digitifera		4	3				2	2			4	4				2	1	
Acropora divaricata		4					2	2								2	2	
Acropora donei		4					3	3										
Acropora echinata		4																
Acropora florida			2				1				4	4				2	2	
Acropora muricata			4				2									2	2	
Acropora gemmifera		4					2									2	2	

Species	Abrolhos									South West								
	Spr			Sum			Aut			Spr			Sum			Aut		
	s	o	n	d	j	f	m	a	m	s	o	n	d	j	f	m	a	m
Acanthastrea echinata							1											
Acropora abrolhosensis							1											
Acropora aculeus							1											
Acropora acuminata																		
Acropora anthocercis							1											
Acropora aspera							1											
Acropora austera																		
nomen dubium																		
Acropora carduus																		
Acropora cerealis							1											
Acropora clathrata																		
Acropora cytherea																		
Acropora abrotanoides																		
Acropora dendrum																		
Acropora desalwii																		
Acropora digitifera							1											
Acropora divaricata																		
Acropora donei																		
Acropora echinata																		
Acropora florida							1											
Acropora muricata							1											
Acropora gemmifera																		

Species	Kimberley Oceanic									Kimberley									Pilbara									Ningaloo									Abrolhos									South West								
	Spr			Sum			Aut			Spr			Sum			Aut			Spr			Sum			Aut			Spr			Sum			Aut			Spr			Sum			Aut			Spr			Sum			Aut		
	s	o	n	d	j	f	m	a	m	s	o	n	d	j	f	m	a	m	s	o	n	d	j	f	m	a	m	s	o	n	d	j	f	m	a	m	s	o	n	d	j	f	m	a	m	s	o	n	d	j	f	m	a	m
Acropora glauca							2	2			2					2				4					3	3																	1											
Acropora grandis		4	4				1	1																	1				4	4				2	2								1											
Acropora granulosa							2	2																																														
Acropora hoeksemai							2	2																																														
Acropora horrida							1	1																																														
Acropora humilis		2	2				1	1			2	2				2				2					2	2			3	3																								
Acropora hyacinthus		2	2				1	1			2	2				2					4				1	3			4	4				2	2																			
Acropora indonesia		4	4				1	1																																														
Acropora intermedia		2	2				1	1				4								4					2	3			4	4				2	2																			
Acropora kosurini																									3	3																												
Acropora latistella		2	2				1	1			2									2	2				1									2	2								1											
Acropora listeri		2	2				2	2																										3	3																			
Acropora loripes		2	2				2	2			4	2								2					4																													
Acropora lutkeni		2	2				2	2			2																																											
Acropora microclados		2	2				1	1				4								4					2																													
Acropora microphthalma		4	4				1	1				4									4				2																													
Acropora millepora	2	1	1				3	3			3	3				2				4	4				1	3			4	4				2	1																			
Acropora monticulosa		1	1				2	2																																														
Acropora muricata		2	2				1	1			4					4				4	4				2				4	4				2	2																			
Acropora nana							2	2			2					2																		2	2																			
Acropora nasuta		2	2				2	2			4	4				2				4	2				1	2			4	4				2	2								1											
Acropora valida							1	1				2																																										

Species	Kimberley Oceanic									Kimberley									Pilbara									Ningaloo									Abrolhos									South West								
	Spr			Sum			Aut			Spr			Sum			Aut			Spr			Sum			Aut			Spr			Sum			Aut			Spr			Sum			Aut			Spr			Sum			Aut		
	s	o	n	d	j	f	m	a	m	s	o	n	d	j	f	m	a	m	s	o	n	d	j	f	m	a	m	s	o	n	d	j	f	m	a	m	s	o	n	d	j	f	m	a	m	s	o	n	d	j	f	m	a	m
Acropora paniculata		4	4				1	1																																														
Acropora papillare							4	4				4				2									3	3			3	3	2	2		4	4																			
Acropora polystoma	2	2	2				1	1																																													
Acropora pulchra							1	1				4													1																		1											
Acropora robusta		2	2				1	1			4	4				2				2					2				4	4				2	2																			
Acropora roseni																									4	2																												
Acropora samoensis		3	3				1	1			2	2				2				2	2			2	2				4	4				2	2																			
Acropora sarmentosa												2				2									3	3																												
Acropora secale		2	2				2	2			2	2				2									2	3			4	4				2	2																			
Acropora selago							1	1			4	4								4					2	2																	1									3	3	2
Acropora seriata																				4																																		
Acropora solitaryensis																				4					2	2																	1											
Acropora spicifera	3	3				1	1			3	3								4	2	2			2				4	4				2	1								1												
Acropora divaricata																																										1												
Acropora striata												4																																										
Acropora subglabra							2	2																																														
Acropora subulata		2	2				2	2													3																																	
Acropora sukarnoi		3	3				2	2																																														
Acropora tenuis	2	1	1				1	1			4	4				2				4	4				1	2			4	4				2	1								1											
Acropora tortuosa																									2																													
Acropora valida																				4																																		
Acropora valenciennesi		2	2				1	1																										2	2																			

Species	Kimberley Oceanic									Kimberley									Pilbara								
	Spr			Sum			Aut			Spr			Sum			Aut			Spr			Sum			Aut		
	s	o	n	d	j	f	m	a	m	s	o	n	d	j	f	m	a	m	s	o	n	d	j	f	m	a	m
Acropora valida		2	2				1	1				4								4	2				2		
Acropora vaughani		2	2				3	3																	3	3	
Acropora verweyi																				4	4				2	3	
Acropora willisae																											
Acropora yongei																											
Alveopora gigas																											
Astreopora gracilis																					4				1		
Astreopora myriophthalma																									2		
Alveopora verrilliana																											
Dipsastraea amicorum																											
Blastomussa wellsi																											
Caulastraea furcata																				4					2		
Caulastraea tumida																					4				4		
Coscinaraea columna																									4		
Coscinaraea exesa																				4					4		
Coscinaraea mcneilli																											
Ctenactis crassa																				4							
Cyphastrea chalcidicum																									2		
Cyphastrea microphthalma																				4							
Cyphastrea serailia																					4				2		
nomen dubium																											
Diploastrea heliopora		1	1																						4		

Species	Ningaloo									Abrolhos									South West								
	Spr			Sum			Aut			Spr			Sum			Aut			Spr			Sum			Aut		
	s	o	n	d	j	f	m	a	m	s	o	n	d	j	f	m	a	m	s	o	n	d	j	f	m	a	m
Acropora valida		4	4				2	2								1											
Acropora vaughani																											
Acropora verweyi																											
Acropora willisae																1											
Acropora yongei																										1	
Alveopora gigas																1											
Astreopora gracilis																											
Astreopora myriophthalma																1											
Alveopora verrilliana																1											
Dipsastraea amicorum																								2			
Blastomussa wellsi																2											
Caulastraea furcata																											
Caulastraea tumida																											
Coscinaraea columna																											
Coscinaraea exesa																											
Coscinaraea mcneilli																								2			
Ctenactis crassa																											
Cyphastrea chalcidicum																											
Cyphastrea microphthalma																1											
Cyphastrea serailia																1											
nomen dubium																									3		
Diploastrea heliopora																											

Species	Kimberley Oceanic									Kimberley									Pilbara									Ningaloo									Abrolhos									South West								
	Spr			Sum			Aut			Spr			Sum			Aut			Spr			Sum			Aut			Spr			Sum			Aut			Spr			Sum			Aut			Spr			Sum			Aut		
	s	o	n	d	j	f	m	a	m	s	o	n	d	j	f	m	a	m	s	o	n	d	j	f	m	a	m	s	o	n	d	j	f	m	a	m	s	o	n	d	j	f	m	a	m	s	o	n	d	j	f	m	a	m
Echinophyllia aspera		4	4				2	2																					4	4				2	2								1											
nomen dubium							2	2																					4	4			1	1	1																			
Echinopora lamellosa							1	1																																														
Euphyllia divisa																																											1											
Euphyllia glabrescens																									2																													
Favites complanata																																											1											
Dipsastraea danai							2	2																																														
Dipsastraea favus																									2																		1											
Dipsastraea maritima																																											1											
Dipsastraea matthaii							2	2																										2	2																			
Dipsastraea maxima																																											1											
Dipsastraea pallida		2	2				2	2				4								4					2									2	2								1											
Dipsastraea rotumana																									2																													
Favites rotundata																									4																													
Dipsastraea speciosa		4	4				1	1																	2																													
Goniastrea stelligera		1	1				1	1				4				2									2																													
Dipsastraea truncata		2	2				2	2																																														
Dipsastraea veroni																									2																													
Favites abdita		1	1				1	1				4				2				4	4				2																		1											
Oulophyllia bennettae																																											1											
Favites flexuosa																				2					2																		1											
Favites halicora		3	3				1	1				4												4	2									2	2								1											

Species	Kimberley Oceanic									Kimberley									Pilbara									Ningaloo									Abrolhos									South West								
	Spr			Sum			Aut			Spr			Sum			Aut			Spr			Sum			Aut			Spr			Sum			Aut			Spr			Sum			Aut			Spr			Sum			Aut		
	s	o	n	d	j	f	m	a	m	s	o	n	d	j	f	m	a	m	s	o	n	d	j	f	m	a	m	s	o	n	d	j	f	m	a	m	s	o	n	d	j	f	m	a	m	s	o	n	d	j	f	m	a	m
Favites pentagona																				4	3	4			2	2																												
Paragoniastrea russelli		4	4				2	2																																			2											
Fungia fungites																				4					2																													
Galaxea astreata							1	1																																														
Galaxea fascicularis							1	1													4				2																		1											
Coelastrea aspera							2	2				4													2																										1	1		
Paragoniastrea australensis																				4				4	2	2																	1						3	1	1			
Goniastrea edwardsi	3						1	1																	2																													
Goniastrea favulus							1	1				4								4					2	2																												
Goniastrea minuta							1	1																																														
Coelastrea palauensis																									2																										4	4		
Goniastrea pectinata							2	2				4								4					2																		1											
Goniastrea retiformis		2	2				1	1				4				2				4	4				2	2			4	4				1	1																			
Goniopora djiboutiensis																																											1											
Goniopora pendulus																																																			2			
Goniopora tenuidens																									4																													
Hydnophora exesa		4	4									3								4					2				4	4				2	2																			
Hydnophora microconos												4																																										
Hydnophora pilosa												3																																										
Hydnophora rigida		2	2				2	2				3																																										
Leptastrea purpurea																				4																																		
Leptastrea transversa																									2																													

Species	Kimberley Oceanic									Kimberley									Pilbara									Ningaloo									Abrolhos									South West								
	Spr			Sum			Aut			Spr			Sum			Aut			Spr			Sum			Aut			Spr			Sum			Aut			Spr			Sum			Aut			Spr			Sum			Aut			Spr			Sum			Aut		
	s	o	n	d	j	f	m	a	m	s	o	n	d	j	f	m	a	m	s	o	n	d	j	f	m	a	m	s	o	n	d	j	f	m	a	m	s	o	n	d	j	f	m	a	m	s	o	n	d	j	f	m	a	m
Leptoria phrygia																				4	4				2									2	2																			
Lobophyllia corymbosa																									2																													
Lobophyllia flabelliformis												4																																										
Lobophyllia hemprichii		2	2									4								4	4			4	2	2			4	4				2	2								1											
Merulina ampliata		4	4				2	2				4								4	4				2				4	4				1	1	1							1											
Merulina scabricula		4	4				2	2																					4	4				2	2																			
Astrea curta							3	3																	2																		1											
Favites magnistellata																				4	4				2																		1											
Astrea curta																									4																													
Montipora capricornis																													4	4				2	2								1											
Montipora efflorescens																									2																													
Montipora grisea																				4																							1											
Montipora hispida																				4	4				1																													
Montipora hoffmeisteri																																											1											
Montipora mollis																																																			2	3	3	1
Montipora monasteriata																				4																																		
Montipora spumosa																													4	4				2	2																			
Montipora tuberculosa																				4																																		
Montipora turgescens																																											1											
Montipora undata																			4	4	4	3	3		2	2																												
Montipora venosa																									4																													
Moseleya latistellata																					4				2																													

Species	Kimberley Oceanic									Kimberley									Pilbara									Ningaloo									Abrolhos									South West								
	Spr			Sum			Aut			Spr			Sum			Aut			Spr			Sum			Aut			Spr			Sum			Aut			Spr			Sum			Aut			Spr			Sum			Aut		
	s	o	n	d	j	f	m	a	m	s	o	n	d	j	f	m	a	m	s	o	n	d	j	f	m	a	m	s	o	n	d	j	f	m	a	m	s	o	n	d	j	f	m	a	m	s	o	n	d	j	f	m	a	m
Mycedium elephantotus												4													2																		1											
Mycedium mancaoi							2	2																																														
Oulastrea crispata		4	4																						2																													
Oxypora lacera																																											1											
Pachyseris speciosa		4	4				2	2																																			1											
Pavona decussata												4							4	4	4	4	4	3	1	2	4																											
Pectinia lactuca																									2																													
Platygyra daedalea							1	1				4				2				4	4				2				4	4				1	1	1																		
Platygyra lamellina																									2																		1											
Platygyra pini							2	2				4													2									2	2																			
Platygyra ryukyuensis							2	2												4					2																													
Platygyra sinensis												4								4	4				2																													
Platygyra verweyi																				4					2																													
Plesiastrea versipora																									2																		1			2	3	3						
Pocillopora damicornis																																														4	4	4	2	2	2	2	2	4
Podabacia crustacea							2	2																																														
Porites cylindrica																									2																													
Porites heronensis																					4				4																													
Porites lobata																			4	4	3	2	4		4																													
Porites lutea																			4	4	4	1	4		4																											1	1	1
Porites (massive)	2	2	2	2	2	2	2	2	2																																													
Symphyllia recta												4													2																													

Species	Kimberley Oceanic									Kimberley									Pilbara									Ningaloo									Abrolhos									South West								
	Spr			Sum			Aut			Spr			Sum			Aut			Spr			Sum			Aut			Spr			Sum			Aut			Spr			Sum			Aut			Spr			Sum			Aut		
	s	o	n	d	j	f	m	a	m	s	o	n	d	j	f	m	a	m	s	o	n	d	j	f	m	a	m	s	o	n	d	j	f	m	a	m	s	o	n	d	j	f	m	a	m	s	o	n	d	j	f	m	a	m
Symphyllia wilsoni																																																			1	1		
Turbinaria mesenterina																			4	4	3	2	2	1	1	1	4																2								2	2	3	
Turbinaria peltata																									4																		1								2	3	2	
Turbinaria reniformis																																																						1
