# Supplementary material for: Coral reproduction in Western Australia
Source: PeerJ. 2016 May 18;4:e2010. doi: 10.7717/peerj.2010 (PMC4878369; doi:10.7717/peerj.2010)
Supplement: Supplemental Information 4 [file peerj-04-2010-s004.pdf]

SUPPLEMENTARY TABLE 3. TAXONOMIC REVISIONS.

There have been a number of significant taxonomic revisions of hard corals in recent years (Arrigoni et al. 2014; Benzoni et al. 2007; Budd et al. 2012; Hoeksema 1989; Huang et al. 2014; Huang et al. 2011; Kitano et al. 2014; Wallace 1999; Wallace et al. 2012). Subsequently, species and genera have been synonymised and re-classified. Reproductive data in Supplementary Table 2 are based on species names used in the original literature, but have been updated here according to the World Register of Marine Species (WORMS) (Appeltans et al. 2012).

Reference name	WORMS nomenclature	Authority
Acropora formosa	*Acropora muricata*	(Linnaeus 1758)
Acropora danai	*Acropora abrotanoides*	(Lamarck 1816)
Acropora stoddarti	*Acropora divaricata*	(Dana 1846)
Acropora tumida	*Acropora valida*	(Dana 1846)
Alveopora fenestrata	*Acropora yongei*	(Veron and Wallace 1984)
Barabattoia amicorum	*Dipsastraea amicorum*	(Milne Edwards & Haime 1849)
Echinophyllia lamellosa	*nomen dubium*	nomen dubium
Favia chinensis	*Euphyllia glabrescens*	(Chamisso and Eysenhardt 1821)
Favia danae	*Dipsastraea danai*	(Milne Edwards 1857)
Favia favus	*Dipsastraea favus*	(Forskål 1775)
Favia maritima	*Dipsastraea maritima*	(Nemezo 1971)
Favia matthaii	*Dipsastraea matthaii*	(Vaughan 1918)
Favia maxima	*Dipsastraea maxima*	(Veron, Pichon & Wijsman-Best 1977)
Favia pallida	*Dipsastraea pallida*	(Dana 1846)
Favia rotumana	*Dipsastraea rotumana*	(Gardiner 1899)
Favia speciosa	*Dipsastraea speciosa*	(Dana 1846)
Favia stelligera	*Goniastrea stelligera*	(Dana 1846)
Favia truncatus	*Dipsastraea truncata*	(Veron 2000)
Favia veroni	*Dipsastraea veroni*	(Moll & Best 1984)
Favites bennettae	*Oulophyllia bennettae*	(Veron Pichon & Best 1977)
Favites russelli	*Paragoniastrea russelli*	(Wells 1954)
Goniastrea australensis	*Paragoniastrea australensis*	(Milne Edwards 1857)
Montastraea curta	*Astrea curta*	(Dana 1846)
Montastraea magnistellata	*Favites magnistellata*	(Chevalier 1971)
Montastrea curta	*Astrea curta*	(Dana 1846)
Mycedium mankaoi	*Mycedium mancaoi*	(Nemenzo 1979)

REFERENCES

Appeltans W, Bouchet P, Boxshall GA, De Broyer C, de Voogd NJ, Gordon DP, Hoeksema BW, Horton T, Kennedy M, Mees J, Poore GCB, Read G, Stöhr S, Walter TC, and MJ. C. 2012. World Register of Marine Species. Accessed at http://www.marinespecies.org on 2013-07-16.

Arrigoni R, Terraneo TI, Galli P, and Benzoni F. 2014. Lobophylliidae (Cnidaria, Scleractinia) reshuffled: pervasive non-monophyly at genus level. *Molecular Phylogenetics and Evolution* 73:60-64.

Benzoni F, Stefani F, Stolarski J, Pichon M, Mitta G, and Galli P. 2007. Debating phylogenetic relationships of the scleractinian Psammocora: molecular and morphological evidences. . *Contributions to Zoology* 76:35-54.

Budd AF, Fukami H, Smith ND, and Knowlton N. 2012. Taxonomic classification of the reef coral family Mussidae (Cnidaria: Anthozoa: Scleractinia). *Zoological Journal of the Linnean Society* 166:465-529.

Hoeksema BW. 1989. Taxonomy, phylogeny and biogeography of mushroom corals (Scleractinia: Fungiidae). *Zoologische Verhandelingen* 254:1-129.

Huang D, Benzoni F, Fukami H, Knowlton N, Smith ND, and Budd AF. 2014. Taxonomic classification of the reef coral families Merulinidae, Montastraeidae, and Diploastraeidae (Cnidaria: Anthozoa: Scleractinia). *Zoological Journal of the Linnean Society* 171:277-355.

Huang D, Licuanan WY, Baird AH, and Fukami H. 2011. Cleaning up the'Bigmessidae': Molecular phylogeny of scleractinian corals from Faviidae, Merulinidae, Pectiniidae and Trachyphylliidae. *Bmc Evolutionary Biology* 11:37.

Kitano YF, Benzoni F, Arrigoni R, Shirayama Y, Wallace CC, and Fukami H. 2014. A Phylogeny of the Family Poritidae (Cnidaria, Scleractinia) Based on Molecular and Morphological Analyses. *Plos one* 9:e98406.

Wallace CC. 1999. *Staghorn corals of the world : a revision of the coral genus Acropora*. Collingwood, Vic.: CSIRO Publishing.

Wallace CC, Done BJ, and Muir PR. 2012. Revision and Catalogue of Worldwide Staghorn Corals *Acropora* and *Isopora* (Scleractina: Acroporidae) in the Museum of Tropical Queensland. Queensland Museum
