# Supplementary material for: Coral reproduction in Western Australia
Source: PeerJ. 2016 May 18;4:e2010. doi: 10.7717/peerj.2010 (PMC4878369; doi:10.7717/peerj.2010)
Supplement: Supplemental Information 5 [file peerj-04-2010-s005.pdf]

SUPPLEMENTARY TABLE 4. REPRODUCTIVE OUTPUT AT A HYPOTHETICAL OCEANIC AND INSHORE REEF OF WESTERN AUSTRALIA

Percentage cover and reproductive data were applied to 17 coral groups that together accounted for most (90%) of the total coral cover at each reef. Quantification of community composition was based on percentage cover estimates derived from replicate sites and habitats within the region of interest. The relative abundances of coral groups were calculated as the percentage cover of each group divided by the total coral cover, and are relevant until disturbances or succession cause major changes in composition. Quantification of the monthly reproductive output was based on the random sampling of species and colonies within each coral group, which weights/biases estimates for the group according to the relative abundance of the species within. The times of reproductive output for spawning corals were inferred from a combination of presence of eggs of different development stages following visual inspection of fragments *in situ* and with a dissection microscope, and some observations of coral spawning; the times of planulae release by brooding corals were based on histological analyses revealing the presence of gametes of varying stages of development and the presence of planulae larvae in visual assessments of colony fragments *in situ* and with a dissection microscope. The reproductive output of each coral group during each month was multiplied by its relative cover within the community. The reproductive output for each coral group during each month was then summed to calculate the total monthly reproductive output. The approach is aimed at identifying the months in which significant reproductive output occurs at the scale of the entire community, and more detailed temporal sampling within these months is required to determine the nights of spawning and planula release, relative to the phases of the moon.

Oceanic Reef

Coral Group	Reproductive	Community Structucture		Percentage reproducing														Proportion reproductive output												
	Mode	Abs. cover	Rel. cover	S	O	N	D	J	F	M	A	M	J	J	A	Total		S	O	N	D	J	F	M	A	M	J	J	A	Total
Poritidae																														
Massive *Porites*	Spawner	12	0.1	0	10	15	15	15	15	15	10	5	0	0	0	100		0.0	1.3	2.0	2.0	2.0	2.0	2.0	1.3	0.7	0.0	0.0	0.0	
Acroporidae																														
Encrusting *Montipora*	Spawner	15	0.2	0	0	0	0	0	0	95	5	0	0	0	0	100		0.0	0.0	0.0	0.0	0.0	0.0	15.8	0.8	0.0	0.0	0.0	0.0	
Branching / Hispidose *Acropora*	Spawner	5	0.1	0	5	0	0	0	0	90	5	0	0	0	0	100		0.0	0.3	0.0	0.0	0.0	0.0	5.0	0.3	0.0	0.0	0.0	0.0	
Corymbose / Digitate *Acropora*	Spawner	11	0.1	0	20	2	0	0	0	75	3	0	0	0	0	100		0.0	2.4	0.2	0.0	0.0	0.0	9.2	0.4	0.0	0.0	0.0	0.0	
Table *Acropora*	Spawner	15	0.2	0	30	2	0	0	0	65	3	0	0	0	0	100		0.0	5.0	0.3	0.0	0.0	0.0	10.8	0.5	0.0	0.0	0.0	0.0	
Faviidae																														
Favia	Spawner	2	0.0	0	20	5	0	0	0	70	5	0	0	0	0	100		0.0	0.4	0.1	0.0	0.0	0.0	1.6	0.1	0.0	0.0	0.0	0.0	
Goniastrea	Spawner	5	0.1	0	0	0	0	0	0	90	10	0	0	0	0	100		0.0	0.0	0.0	0.0	0.0	0.0	5.0	0.6	0.0	0.0	0.0	0.0	
Favites	Spawner	2	0.0	0	10	5	0	0	0	80	5	0	0	0	0	100		0.0	0.2	0.1	0.0	0.0	0.0	1.8	0.1	0.0	0.0	0.0	0.0	
Playtgyra	Spawner	2	0.0	0	0	0	0	0	0	90	10	0	0	0	0	100		0.0	0.0	0.0	0.0	0.0	0.0	2.0	0.2	0.0	0.0	0.0	0.0	
Echinopora	Spawner	2	0.0	0	0	0	0	0	0	90	10	0	0	0	0	100		0.0	0.0	0.0	0.0	0.0	0.0	2.0	0.2	0.0	0.0	0.0	0.0	
Pocilloporidae																														
Pocillopora	Spawner	2	0.0	0	25	25	0	0	0	25	25	0	0	0	0	100		0.0	0.6	0.6	0.0	0.0	0.0	0.6	0.6	0.0	0.0	0.0	0.0	
Agariciidae																														
Pavona	Spawner	0	0.0	0	0	0	0	0	0	40	60	0	0	0	0	100		0.0	0.0	0.0	0.0	0.0	0.0	0.0	0.0	0.0	0.0	0.0	0.0	
Dendrophylliidae																														
Turbinaria	Spawner	0	0.0	0	10	10	10	10	10	20	20	10	0	0	0	100		0.0	0.0	0.0	0.0	0.0	0.0	0.0	0.0	0.0	0.0	0.0	0.0	
Acroporidae																														
Isopora	Brooder	10	0.1	5	15	15	10	10	10	15	15	5	0	0	0	100		0.6	1.7	1.7	1.1	1.1	1.1	1.7	1.7	0.6	0.0	0.0	0.0	
Seriatopora	Brooder	3	0.0	5	15	15	10	10	10	15	15	5	0	0	0	100		0.2	0.5	0.5	0.3	0.3	0.3	0.5	0.5	0.2	0.0	0.0	0.0	
Stylophora	Brooder	2	0.0	5	15	15	10	10	10	15	15	5	0	0	0	100		0.1	0.3	0.3	0.2	0.2	0.2	0.3	0.3	0.1	0.0	0.0	0.0	
Pocillopora	Brooder	2	0.0	5	15	15	10	10	10	15	15	5	0	0	0	100		0.1	0.3	0.3	0.2	0.2	0.2	0.3	0.3	0.1	0.0	0.0	0.0	
**Total**		**90**	**1**															**0.9**	**13.1**	**6.2**	**3.9**	**3.9**	**3.9**	**58.6**	**7.9**	**1.6**	**0.0**	**0.0**	**0.0**	**100**

Inshore Reef

Coral Group	Reproductive	Community Strucuture		Percentage reproducing														Proportion reproductive output												
	Mode	Abs. cover	Rel. cover	S	O	N	D	J	F	M	A	M	J	J	A	Total		S	O	N	D	J	F	M	A	M	J	J	A	Total
Poritidae																														
Massive *Porites*	Spawner	20	0.2	0	0	5	90	5	0	0	0	0	0	0	0	100		0.0	0.0	1.1	20.0	1.1	0.0	0.0	0.0	0.0	0.0	0.0	0.0	
Acroporidae																														
Encrusting *Montipora*	Spawner	5	0.1	0	0	0	0	0	0	95	5	0	0	0	0	100		0.0	0.0	0.0	0.0	0.0	0.0	5.3	0.3	0.0	0.0	0.0	0.0	
Branching / Hispidose *Acropora*	Spawner	0																												
Corymbose / Digitate *Acropora*	Spawner	5	0.1	0	10	2	0	0	0	78	10	0	0	0	0	100		0.0	0.6	0.1	0.0	0.0	0.0	4.3	0.6	0.0	0.0	0.0	0.0	
Table *Acropora*	Spawner	2	0.0	0	10	2	0	0	0	78	10	0	0	0	0	100		0.0	0.2	0.0	0.0	0.0	0.0	1.7	0.2	0.0	0.0	0.0	0.0	
Faviidae																														
Favia	Spawner	5	0.1	0	0	0	0	0	0	95	5	0	0	0	0	100		0.0	0.0	0.0	0.0	0.0	0.0	5.3	0.3	0.0	0.0	0.0	0.0	
Goniastrea	Spawner	5	0.1	0	0	0	0	0	0	95	5	0	0	0	0	100		0.0	0.0	0.0	0.0	0.0	0.0	5.3	0.3	0.0	0.0	0.0	0.0	
Favites	Spawner	5	0.1	0	5	5	0	0	0	80	10	0	0	0	0	100		0.0	0.3	0.3	0.0	0.0	0.0	4.4	0.6	0.0	0.0	0.0	0.0	
Playtgyra	Spawner	5	0.1	0	0	0	0	0	0	95	5	0	0	0	0	100		0.0	0.0	0.0	0.0	0.0	0.0	5.3	0.3	0.0	0.0	0.0	0.0	
Echinopora	Spawner	5	0.1	0	0	0	0	0	0	95	5	0	0	0	0	100		0.0	0.0	0.0	0.0	0.0	0.0	5.3	0.3	0.0	0.0	0.0	0.0	
Pocilloporidae																														
Pocillopora	Spawner	2	0.0	0	10	5	0	0	0	80	5	0	0	0	0	100		0.0	0.2	0.1	0.0	0.0	0.0	1.8	0.1	0.0	0.0	0.0	0.0	
Agariciidae																														
Pavona	Spawner	15	0.2	0	0	0	0	0	0	40	60	0	0	0	0	100		0.0	0.0	0.0	0.0	0.0	0.0	6.7	10.0	0.0	0.0	0.0	0.0	
Dendrophylliidae																														
Turbinaria	Spawner	10	0.1	0	10	10	10	10	10	20	20	10	0	0	0	100		0.0	1.1	1.1	1.1	1.1	1.1	2.2	2.2	1.1	0.0	0.0	0.0	
Acroporidae																														
Isopora	Brooder	0	0.0	5	15	15	10	10	15	15	10	5	0	0	0	100		0.0	0.0	0.0	0.0	0.0	0.0	0.0	0.0	0.0	0.0	0.0	0.0	
Seriatopora	Brooder	2	0.0	5	15	15	10	10	15	15	10	5	0	0	0	100		0.1	0.3	0.3	0.2	0.2	0.3	0.3	0.2	0.1	0.0	0.0	0.0	
Stylophora	Brooder	2	0.0	5	15	15	10	10	15	15	10	5	0	0	0	100		0.1	0.3	0.3	0.2	0.2	0.3	0.3	0.2	0.1	0.0	0.0	0.0	
Pocillopora	Brooder	2	0.0	5	15	15	10	10	15	15	10	5	0	0	0	100		0.1	0.3	0.3	0.2	0.2	0.3	0.3	0.2	0.1	0.0	0.0	0.0	
**Total**		**90**	**1**															**0.3**	**3.4**	**3.8**	**21.8**	**2.9**	**2.1**	**48.6**	**15.7**	**1.4**	**0.0**	**0.0**	**0.0**	**100**
